# Impact of Vaccination-Differentiated Public Health and Social Measures on Vaccine Uptake Among the Vaccine Hesitant

**DOI:** 10.1016/j.focus.2025.100419

**Published:** 2025-08-20

**Authors:** En Jie Tan, Cheryl Chong, Alex Yap, Calvin Chiew, Sharon Tan, Yuhan Yang, Kelvin Tan, David Lye, Alex Cook, Vernon Lee

**Affiliations:** 1 Communicable Diseases Division, Ministry of Health, Singapore, Singapore; 2 National Centre for Infectious Diseases, Singapore, Singapore; 3 Saw Swee Hock School of Public Health, National University of Singapore and National University Health System, Singapore, Singapore; 4 Lee Kong Chian School of Medicine, Nanyang Technological University, Singapore, Singapore

**Keywords:** Vaccination hesitancy, vaccination refusal, physical distancing, communicable disease control, public health, attitude to health, COVID-19

## Abstract

**Introduction:**

Vaccine hesitancy poses a hurdle toward achieving high population-level immunity and reducing disease transmission. One policy response adopted during the COVID-19 vaccination campaign was the implementation of vaccination-differentiated public health and social measures. This paper aims to evaluate the impact of vaccination-differentiated public health and social measures on COVID-19 vaccination rates in Singapore, with a specific focus on vaccine-hesitant persons.

**Methods:**

An interrupted time series design with segmented linear regression was utilized to assess the impact of the announcement of each vaccination-differentiated public health and social measure on vaccine uptake rates among the vaccine-naive population. Newey-West standard errors with a 7-day lag were applied to address potential autocorrelation in the data. Covariates adjusted for included day of the week, daily numbers of COVID-19–related cases, deaths, hospitalizations, and patients in the intensive care unit.

**Results:**

The study found a significant and positive effect of vaccination-differentiated public health and social measure announcements on the daily vaccine uptake rate among the vaccine-naive population, specifically for dining out and patronizing shopping malls. When stratified by age group, the significant and positive effect of the announcement of mall access vaccination-differentiated public health and social measures persisted across all age groups, and it only persisted for individuals aged 13–17, 25–34, and 35–49 years for the announcement of dining vaccination-differentiated public health and social measures.

**Conclusions:**

Overall, the findings support the effectiveness of vaccination-differentiated public health and social measures in increasing vaccine uptake rates among vaccine-hesitant persons. However, the compelling evidence for complementary policies should also be considered.

## Highlights

• Vaccine hesitancy poses a hurdle toward achieving population vaccine coverage.
• One policy response adopted for vaccine hesitancy was vaccination-differentiated public health and social measures (VDMs).
• VDMs for dining out and shopping malls proved effective among the vaccine-naïve population.
• Dining VDMs, in particular, proved most effective among younger age groups.

## INTRODUCTION

The coronavirus disease 2019 (COVID-19) pandemic highlighted the importance of vaccination in reducing viral transmission, hospitalization, severe disease, and mortality.^1^^,^^2^ Despite the expedient nature of vaccine development, COVID-19 vaccines upheld safety standards that were noninferior to those of routine viral vaccines,^3^, ^4^, ^5^, ^6^, ^7^ thereby providing a solution with a favorable risk–benefit profile to the ongoing pandemic for the general and at-risk populations alike.^8^, ^9^, ^10^

Substantial scholarship has investigated trends in COVID-19 vaccination rates and the impact of policy and nonpolicy measures on vaccine uptake among the populace, including open data sharing, evidence-based medicine behind policy making, combating disinformation, and vaccine mandates.^11^, ^12^, ^13^ Nevertheless, isolating the incremental impact of provaccination policies from the inherent willingness of the population to get vaccinated proved to be complex, particularly during the early phases of vaccination when vaccine acceptance, enthusiasm, and motivation were high in the majority of the population.^14^, ^15^, ^16^, ^17^ As the pandemic progressed, a greater understanding of this relationship in communities with suboptimal vaccine uptake became an area of growing interest both within public health circles and within the academic community.

**Figure 1:**
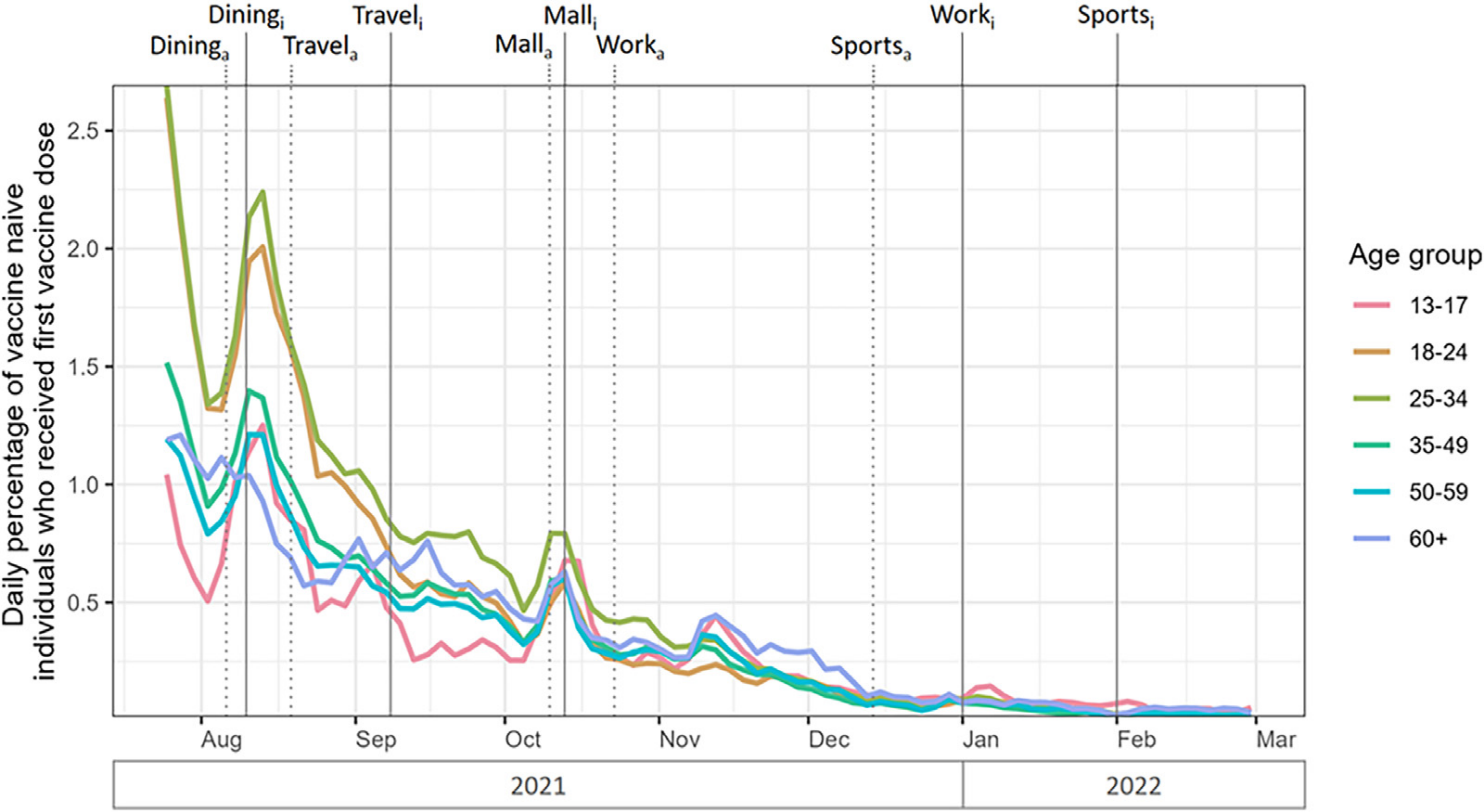
Daily percentage of vaccine-naive individuals among the medically eligible population who received their first vaccine dose, by age group, from July 25, 2021 to February 28, 2022. *Note*: For each VDM, announcement dates were indicated by subscript “a” and dotted vertical lines, whereas implementation dates were indicated by subscript “i” and solid vertical lines. VDM, vaccination-differentiated public health and social measure.

Vaccine hesitancy, defined as a delay in acceptance or refusal of safe vaccines despite the availability of vaccination services,^18^ was highlighted by the WHO as 1 of the top 10 threats to global health.^19^ Reasons for hesitancy are varied, including concerns about vaccine safety and efficacy,^20^, ^21^, ^22^, ^23^ a perceived low risk of infection,^24^ limited access to healthcare services, and strong personal convictions against vaccination,^25^ coined by Fournet et al. as the hesitant, unconcerned, poorly reached, and active resistors, respectively. In general, vaccine hesitancy poses a hurdle toward achieving high population-level immunity,^26^, ^27^, ^28^, ^29^, ^30^ which is important not only for personal protection but also for the effect of collective protection by reducing disease transmission. For COVID-19, vaccination is particularly important for the elderly population, who are at increased risk for both infection, severe illness, and mortality yet have higher vaccine hesitancy.^31^, ^32^, ^33^, ^34^, ^35^, ^36^, ^37^ Singapore was quick to attain high rates of vaccination (Appendix Figure 1, available online), although 15.4% of the population remained vaccine hesitant, opting not to get vaccinated despite their eligibility and the widespread availability of vaccines (Figure 1).

One policy response adopted during the COVID-19 vaccination campaign was the implementation of vaccination-differentiated public health and social measures (VDMs).^38^, ^39^, ^40^ VDMs granted only individuals who received COVID-19 vaccinations with freedom for social interaction and travel, thereby bifurcating the number of contacts of vaccine-protected and susceptible subpopulations. Persons who were medically ineligible for vaccination or recovered from COVID-19 within the last 270 days were excluded from such restrictions. Similar terms for such policies included COVID-19 vaccine passports, vaccine certificates, health passes, green passes, and vaccine mandates. In Singapore, 5 sequential VDMs were progressively implemented for dining, travel, malls access, work, and sports facilities/institutes of higher learning (IHLs)/hotels in the latter half of the COVID-19 pandemic from the period of August 10, 2021 to October 10, 2022. Significant prior initiatives to increase vaccination included instilling public confidence in vaccines through vaccination of prominent public figures (January 21, 2021), a financial assistance program for persons with postvaccination complications (March 17, 2021), vaccination on a walk-in basis (June 1, 2021), and home vaccination for elderly persons (July 15, 2021). Although the effects of implementing VDMs on increasing overall vaccination rates have been established for the general population,^22^^,^^34^^,^^41^, ^42^, ^43^, ^44^, ^45^, ^46^, ^47^, ^48^, ^49^, ^50^ their effectiveness has yet to be explored for the vaccine-hesitant subpopulation. Understanding this influence is beneficial for informing future policy decisions and optimizing the response to ongoing and emerging pandemics. This paper therefore aims to evaluate the impact of VDMs on COVID-19 vaccination rates in Singapore, with a specific focus on vaccine-hesitant persons.

## METHODS

### Study Sample

Vaccine-hesitant persons were defined in this study as persons in Singapore who remained COVID-19 vaccine naive in the second half of the pandemic (from July 25, 2021 onward) when vaccines had become widely available. Vaccine uptake was defined as the daily percentage of vaccine-naive Singaporeans and permanent residents aged ≥13 years who received their first dose of the COVID-19 vaccine. Persons aged ≤12 years were excluded from this study because COVID-19 vaccines were only introduced for this age group during the implementation of VDMs in Singapore. The vaccine rollout schedule is provided in Appendix Table 1 (available online).

Retrospective population-wide vaccination data were obtained from the Ministry of Health (MOH) because COVID-19 vaccinations were reportable by law to the MOH under the Infectious Diseases Act. Daily vaccination rates were obtained from the period of July 1, 2021, to February 28, 2022. This study did not require IRB/ethical approval.

### Measures

An interrupted time series (ITS) design with segmented linear regression was utilized to assess the impact of the announcement of each VDM on vaccine uptake rates among the vaccine-naive population.^51^ The 5 key interventions were (1) the announcement of VDM for customers to dine at food and beverage establishments on August 6, 2021; (2) the announcement of VDM for cross-border travel to selected countries (and progressively expanded) on August 19, 2021; (3) the announcement of VDM to patronize shopping malls and large standalone stores on October 10, 2021; (4) the announcement of VDM for returning to office workspaces on October 23, 2021; and (5) the announcement of VDM for entry to and usage of sports facilities, visitation by nonacademic persons to IHLs, and access to guest floors within hotel premises on December 14, 2021.

The data series commenced on July 25, 2021. The segments modeled were July 25, 2021, to August 5, 2021 (baseline); August 6, 2021, to August 18, 2021 (dining VDM); August 19, 2021, to October 9, 2021 (travel VDM); October 10, 2021, to October 22, 2021 (shopping malls VDM); October 23, 2021, to December 13, 2021 (work VDM); and December 14, 2021, to December 26, 2021 (sporting facilities, IHLs, and hotels VDM). Vaccine uptake rates in each period were compared with those in prior periods to estimate level and trend changes. Of the number of vaccine-naive individuals, the proportion that received the first dose of the COVID-19 vaccine was calculated daily. Changes in vaccine uptake rates were assessed at the point of announcement of the VDMs because studies in France, Italy, Israel, and Switzerland suggested that the anticipation of such measures alone serves as a significant motivator for vaccination^44^ and a stronger motivator for vaccination than the implementation of VDMs.^13^^,^^52^

### Statistical Analysis

Newey-West SEs with a 7-day lag were applied to address potential autocorrelation in the data^53^ (Appendix Figures 2–5, available online). Sensitivity analyses were conducted by excluding individuals with prior COVID-19 infections within the past 270 days as well as individuals with recorded allergies to the COVID-19 vaccine. Additional covariates (day of the week, daily numbers of COVID-19–related cases, deaths, hospitalizations, and patients in the intensive care unit [ICU] as reported by MOH) were also adjusted for. Further sensitivity checks were carried out using VDM implementation dates instead of announcement dates as key time points and by adopting distinct ITS models for each VDM announcement. The analysis was performed using R, Version 4.3.1.

## RESULTS

Over the course of VDM announcements, the number of individuals who were eligible for vaccination but remained unvaccinated decreased from 603,717 (16.1% of the population eligible for vaccination) on July 25, 2021—12 days before the first announcement date—to 265,739 (7.1%) at December 26, 2021—12 days after the last announcement.

**Figure 2:**
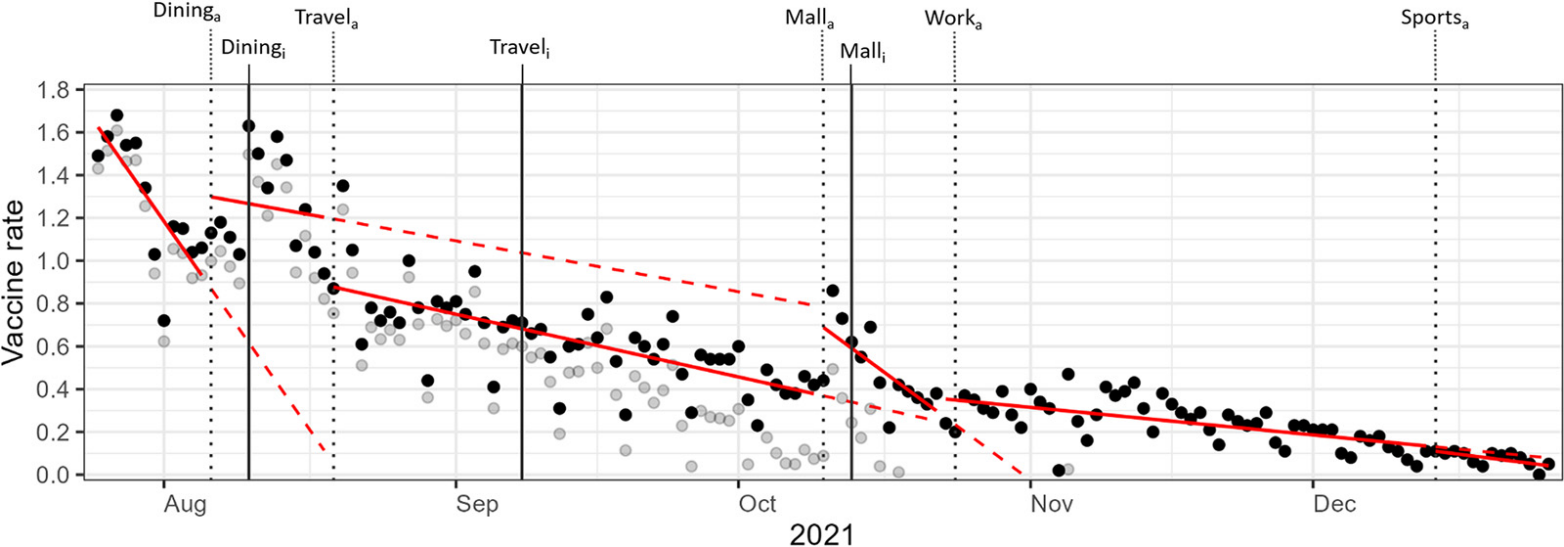
Fitted lines of unadjusted segmented regression model for daily vaccine uptake rate. *Note*: Opaque points represent the daily vaccine uptake rate from July 25, 2021, to February 13, 2022. Translucent points represent the vaccine uptake rate after adjusting for effects of the day of the week and daily reported numbers of COVID-19–related infections, hospitalizations, ICU cases, and deaths. For each VDM, announcement dates are indicated by subscript “a” and dotted vertical lines, whereas implementation dates are indicated by subscript “i” and solid vertical lines (specific dates are detailed in Appendix Table 4, available online). Solid fitted lines in red represent the unadjusted segmented regression model of vaccine uptake rate at each period. Dashed fitted lines in red represent the expected vaccine uptake rate on the basis of the preceding period had the intervention not occurred. ICU, intensive care unit; VDM, vaccination-differentiated public health and social measure.

Figure 2 illustrates the fitted lines of the unadjusted segmented regression model for the daily vaccine uptake rate, from July 25, 2021, to December 26, 2021. An increase in vaccine uptake was observed after the VDM announcements for dining at food and beverage establishments, patronizing shopping malls and large standalone stores, and returning to office workspaces but not for cross-border travel and entry to and usage of sports facilities, IHLs, and hotels.

Results of the segmented linear regression model are presented in Table 1. Effects of the VDM announcements were measured as percentage-point changes in daily vaccine uptake rates. The model found a significant and positive effect of VDM announcements on the daily vaccine uptake rate among the vaccine-naive population, specifically for dining out (β=0.41, 95% CI=0.05, 0.76, *p*=0.027) and patronizing shopping malls (β=0.31, 95% CI=0.22, 0.40, *p*≤0.001) (Table 1). No statistically significant effects were observed for the other VDMs.

**Table 1:** Results of the Segmented Linear Regression Model, Describing Percentage-Point Changes (95% CIs) in Daily Vaccine Uptake Rates After Each VDM Announcement, for the Overall Population and by Age Group

Age group, years	Dining	Travel	Malls	Work	Sports/IHLs/hotels
Overall	0.41 (0.05, 0.76)*	−0.31 (−0.63, 0.01)	0.31 (0.22, 0.40)***	0.09 (−0.01, 0.20)	−0.02 (−0.06, 0.03)
13–17	0.69 (0.35, 1.03)***	−0.23 (−0.81, 0.35)	0.43 (0.12, 0.74)**	0.02 (−0.16, 0.20)	−0.04 (−0.10, 0.01)
18–24	0.69 (−0.00, 1.37)	−0.60 (−1.30, 0.11)	0.44 (0.28, 0.60)***	0.07 (0.02, 0.11)**	0.01 (−0.02, 0.03)
25–34	0.69 (0.01, 1.37)*	−0.69 (−1.37, −0.01)*	0.42 (0.30, 0.54)***	0.10 (0.02, 0.18)*	0.03 (0.02, 0.05)***
35–49	0.42 (0.03, 0.81)*	−0.31 (−0.68, 0.06)	0.34 (0.25, 0.43)***	0.09 (−0.01, 0.19)	0.00 (−0.03, 0.03)
50–59	0.28 (−0.07, 0.63)	−0.33 (−0.62, −0.04)*	0.33 (0.24, 0.42)***	0.14 (0.01, 0.27)*	−0.02 (−0.06, 0.03)
≥60	0.13 (−0.08, 0.33)	0.01 (−0.13, 0.15)	0.16 (0.02, 0.29)*	0.07 (−0.09, 0.23)	−0.08 (−0.17, 0.02)

*Note*: *p<0.05, **p<0.01, and ***p<0.001.

IHL, institute of higher learning; VDM, vaccination-differentiated public health and social measure.

When stratified by age group, the significant and positive effect of the announcement of mall access VDMs persisted across all age groups. Greater effect sizes were observed in the younger age groups (ages 13–17 years: β=0.43, 95% CI=0.12, 0.74, *p*=0.006; ages 18–24 years: β=0.44, 95% CI=0.28, 0.60, *p≤*0.001; ages 25–34 years: β=0.42, 95% CI=0.30, 0.54, *p*≤0.001), followed by those in middle age (ages 35–49 years: β=0.34, 95% CI=0.25, 0.43, *p*≤0.001; ages 50–59 years: β=0.33, 95% CI=0.24, 0.42, *p*≤0.001), whereas older adults aged ≥60 years had smaller effect sizes (β=0.16, 95% CI=0.02, 0.29, *p*=0.02).

The significant and positive effect of the announcement of dining VDMs persisted for individuals aged 13–17 years (β=0.69, 95% CI=0.35, 1.03, *p*≤0.001), those aged 25–34 years (β=0.69, 95% CI=0.01, 1.37, *p*=0.048), and those aged 35–49 years (β=0.42, 95% CI=0.03, 0.81, *p*=0.035) but not for the other age groups.

A significant increase in daily vaccine uptake rate was also observed after the announcement of work VDMs for those aged 18–24 years (β=0.07, 95% CI=0.02, 0.11, *p*=0.005), those aged 25–34 years (β=0.10, 95% CI=0.02, 0.18, *p*=0.011), and those aged 50–59 (β=0.14, 95% CI=0.01, 0.27, *p*=0.031). For those aged 25–34 years, there was an additional effect of the VDM announcement for entry to and usage of sports facilities/IHLs/hotel stays (β=0.03, 95% CI=0.02, 0.05, *p*≤0.001).

Vaccination uptake rates were reported alongside trends in COVID-19–related cases, deaths, hospitalizations, and patients in the ICU (Appendix Figures 1 and 6–9, available online) and vaccination rollout schedules (Appendix Table 1, available online). Results of sensitivity analyses (Table 2 and Appendix Table 2, available online) indicated that the announcement of VDM for dining and mall access had a positive effect on daily vaccine uptake rates, consistent with findings from the main model. A positive effect (β=0.36, 95% CI=0.17, 0.55, *p*≤0.001) was also observed for the dining VDM when assessed by policy implementation date (Appendix Table 3, available online).

**Table 2:** Results of the Sensitivity Analyses, Describing Percentage-Point Changes (95% CIs) in Daily Vaccine Uptake Rates After Each VDM Announcement for Each Model.

Model	Dining	Travel	Malls	Work	Sports/IHLs/hotels
Segmented regression, with exclusions^a^	0.43 (0.05, 0.81)*	−0.32 (−0.66, 0.02)	0.32 (0.23, 0.41)***	0.09 (−0.02, 0.19)	−0.02 (−0.07, 0.03)
Segmented regression, adjusted^b^	0.43 (0.12, 0.74)**	−0.24 (−0.58, 0.10)	0.36 (0.29, 0.42)***	0.12 (0.04, 0.20)**	0.03 (−0.04, 0.10)

*Note*: *p<0.05, **p<0.01, and ***p<0.001.

ICU, intensive care unit; IHL, institute of higher learning; MOH, Ministry of Health; VDM, vaccination-differentiated public health and social measure.

a Individuals with prior COVID-19 infections within the past 270 days or who had recorded allergies to the COVID-19 vaccine were excluded from the vaccine-eligible pool each day.

b Adjusted for day of the week, daily numbers of COVID-19–related cases, deaths, hospitalizations, and patients in the ICU as reported by the MOH.

## DISCUSSION

Of the 5 sequential VDMs implemented in Singapore, VDM announcements for mall access and dining were associated with increasing vaccine uptake rates for the overall population, with the VDM for mall access demonstrating a consistent effect across all age subgroups. These results, along with those of the sensitivity analysis for VDM implementation dates (Appendix Table 3, available online), support existing literature that vaccine uptake was largely driven by policy announcements. Although the percentage-point increase in the daily vaccine uptake rate after the announcement of the VDM for mall access was <1%, the practical implications for public health are substantial, with a total of 18,961 first vaccine doses administered during this period (Appendix Table 4, available online).

VDMs were also notably effective in France, Israel, Italy, and Switzerland, where they were enacted for use of hospitals, public transport services, malls, areas of worship, and hospitality services.^44^ These findings may be explained through behavioral economics.^54^^,^^55^ VDMs can be understood through the lens of loss aversion, where the prospect of losing access to desired activities (dining, mall access) serves as a stronger motivator than the potential gain of vaccination itself. The immediate restriction on activities taps into present bias, making the immediate consequence of nonvaccination more salient than long-term health benefits.^43^^,^^56^^,^^57^ Considering the theory of planned behavior, possible mechanisms behind this behavioral change include the redefining of vaccination as a social norm through VDMs^58^^,^^59^ and increased perceived control of vaccination through public communications on actions to circumvent the consequences of VDMs.

Study findings differed from those of other studies that suggested that VDMs had limited positive effects on countries with high vaccine uptakes^44^ and that there was limited efficacy of VDMs to incentivize vaccination for reasons other than travel.^60^ Similar to those in Singapore, VDMs in the countries where these studies were conducted allowed for negative polymerase chain reaction or antigen rapid test swabs for a temporary, time-limited lifting of restrictions in place of vaccination.

Notably, a smaller magnitude of percentage increase in vaccine uptake after VDMs was observed in this study than prior literature. In Singapore, VDMs were introduced in succession, thereby likely underestimating the true effect of each VDM if it were introduced independently. In addition, VDMs were only rolled out when vaccination rates already exceeded 80% of the population. The findings in this study only represented the effects on the remaining 20% of the vaccine-hesitant population for the period from August 2021 to February 2022. Conversely, jurisdictions with greater vaccine uptake after VDMs, such as Denmark, Israel, Italy, France, Germany, and Switzerland, initiated vaccine certification from April to August 2021, when jurisdiction-specific vaccination rates were significantly lower, between 2.67% and 63.1%.^61^

In the subgroup analysis for each VDM by age groups, the greatest responsiveness to VDMs was observed in the younger age groups, particularly for individuals aged 13–34 years, during the dining and mall access VDMs. One possible explanation may be the short lead time of 2 months that Singaporeans, aged 12–39 years, had from their date of eligibility for vaccination till the implementation of VDMs in Singapore, a confounder acknowledged by studies observing VDMs in other jurisdictions as well. Hitherto, this does not detract from the statistically significant increase in first-time-vaccination rates after the announcement of VDMs compared with the baseline prior to the announcement. These results are also consistent with existing literature^43^^,^^56^^,^^62^ on the general population, which suggested that younger people were more susceptible to factors that influenced their social network^63^, ^64^, ^65^ and, therefore, more amenable to VDMs. In another study, this effect was most pronounced when restrictions extended to large social settings such as nightclubs and events with >1,000 people for adolescents and for more intimate social events, the hospitality sector, and leisure activities for adults aged <50 years.^44^

The VDMs for work were associated with increasing vaccine uptake for persons aged 18–34 years and 50–59 years, albeit a modest response compared with VDMs for dining and mall access. The positive impact of this VDM on the working population supported the notion that the prospect of financial disincentives served as a strong motivator for compliance to vaccination.^66^^,^^67^ This phenomenon was likely compounded by the protracted nature of the pandemic, which undermined the job security of many.^68^, ^69^, ^70^, ^71^, ^72^ The effectiveness of VDMs for work was consistent with findings from other studies,^73^ some of which purported its superiority over statewide VDMs,^74^^,^^75^ particularly when there was alignment between government and employer vaccination goals.^76^

Further subgroup analysis was considered between ideological resistors and persons with access-related barriers for vaccination. However, several factors limit the plausibility of access barriers for vaccination, including the availability of vaccines from hospitals, community-based testing and vaccination facilities, and general practitioner clinics; the absence of vaccine supply limitations at the time of the study; and home-based vaccination programs.

A possible explanation for the diminished impact that VDMs for work had was their announcement in close succession to the VDM for mall access. VDMs for dining and mall access showed a statistically significant increase in vaccination rates, but those announced shortly after (travel and work, respectively) proved to have a lesser impact. This suggested a first-mover advantage that waned with subsequent announcements, particularly those in close succession. To account for this, we separated the observation periods (12 days before and after each VDM) as much as possible to minimize overlaps. A longer window could potentially have yielded more robust results, but it was not feasible in this study. It also remains unclear whether the targeted activity for each VDM or the timing and sequence of VDM measures played a significant role in each VDM’s effectiveness.

In evaluating the blunted effectiveness of the VDM for sport facilities/IHLs/hotel stays, a factor considered was the concentration of persons with strong antivaccination sentiments in the remaining unvaccinated population over the course of VDM implementation. Prior VDMs may have motivated all undecided persons toward vaccination. The remnant antivaccination-dominated population would therefore already have had a strong disposition against vaccination. VDMs have been shown to be ineffective for this demographic.^77^

Another consideration was restriction fatigue of the remaining vaccine-hesitant persons, a corollary to vaccine fatigue^78^^,^^79^ for the general population. Over the course of the pandemic, vaccine-hesitant persons may have grown weary of repeated messaging and campaigns promoting vaccination. This fatigue could exacerbate concerns and strengthen the beliefs of the hesitant, driving them to join the active resistors, as seen by the increased autonomy frustration in Israel after VDMs.^80^ Vaccine fatigue could also manifest as a sense of apathy and shift previously hesitant or poorly reached persons to the unconcerned subgroups^25^ among vaccine-hesitant persons.

This study specifically looked at the vaccine-hesitant population, a crucial subgroup for achieving high vaccination rates and population-wide protection from diseases. The absence of concurrent policies implemented during the study period created ideal conditions for the ITS evaluation of VDM on vaccine-hesitant persons (Appendix Table 5, available online). The ITS design also allowed for impact analysis of VDM announcements on daily vaccination rates among the vaccine-naive population. This approach provided a more nuanced understanding of the immediate and sustained effects of VDMs. By exploring the differential effects of VDMs on various age groups, the analysis further revealed how specific VDMs may resonate more strongly with certain age demographics on the basis of social interaction patterns and priorities. Potential confounding variables, such as daily COVID-19 cases, deaths, hospitalizations, and ICU admissions, were accounted for to strengthen the internal validity of the findings.

Furthermore, the demographics of the vaccine-hesitant population were also evaluated at 2 time points: 12 days before and 12 days after VDMs. Using Cohen’s h as a metric (Appendix Table 6, available online), the changes in demographic proportion are mostly negligible to small, with the exception for those living in private housing (h= −0.3). This suggests that the potential of VDMs for marginalizing groups on the basis of sex, ethnicity, housing type, and age was limited.

### Limitations

Although this study offered valuable insights into the effectiveness of VDMs in the vaccine-hesitant population of Singapore, several limitations should be considered when extrapolating these findings to other populations. This study employed linear regression to model the effect of each successive VDM. Country- and population-specific cultural and societal contexts^81^, ^82^, ^83^ such as the collectivistic nature of Singaporean society, a centralized and accessible^84^, ^85^, ^86^, ^87^ healthcare system, and strong public trust in government^88^ should be considered when crafting policies to increase vaccine uptake. The observed effectiveness of VDMs, particularly those leveraging social exclusion, is likely amplified in societies with strong communitarian values. Conversely, settings with fragmented healthcare systems or lower public trust may face poor public acceptance, potentially undermining the implementation and overall impact of VDMs. The effectiveness of VDMs may also vary depending on their timing and sequence during a pandemic. Similar to other papers examining the effects of VDMs through national-level data,^89^, ^90^, ^91^, ^92^, ^93^ this study was unable to further understand individual-level motivators and deterrents influencing the choice for vaccination. Given the limitations of the data, the authors were unable to explore potential unintended consequences of VDMs, as reported in the literature.

VDMs, although aiming for public health benefits, may also raise ethical concerns regarding personal liberty and individual autonomy. By restricting access to certain public spaces, services, or employment for unvaccinated individuals, such measures can be perceived as limiting fundamental freedoms and the right to make independent health decisions without undue pressure. Furthermore, these policies have the potential to increase inequities, disproportionately affecting vulnerable populations who may face greater barriers to vaccination (e.g., owing to SES, limited access to health care, or historical mistrust), thus exacerbating existing social disparities.^94^ The differentiation between vaccinated and unvaccinated individuals can strain social cohesion^95^, ^96^, ^97^, ^98^ and increase marginalization of certain groups.^99^^,^^100^ These aspects warrant further consideration, particularly against the backdrop of global vaccine discrepancies and inequitable access to testing facilities.

Finally, VDMs may provide a false sense of assurance for the vaccinated population, thereby decreasing infection control practices, including social distancing and hand washing.^101^ Whereas the study attempted to mitigate the waning effect of each announcement through short observation periods, alternative functions may be considered for subsequent vaccine uptake modeling as scholarship expands within this domain.

## CONCLUSIONS

Overall, study findings support the effectiveness of VDMs in increasing vaccine uptake rates among vaccine-hesitant persons. However, the compelling evidence for complementary policies should also be considered, including increasing the ease of accessibility to vaccine services,^102^^,^^103^ public education programs to address safety concerns of vaccines,^104^, ^105^, ^106^, ^107^ programs to target subpopulations with lower vaccine adherence,^46^^,^^108^^,^^109^ role modeling by public health officials,^110^ and vaccine administration by medical practitioners.^43^

Exploring the influence of socioeconomic factors on vaccine uptake among the vaccine-hesitant population, such as sex,^111^, ^112^, ^113^ income level, marital status, education level,^114^, ^115^, ^116^ and subjective factors such as perceived vaccine safety, knowledge of the protection conferred, personal risk perception of infection, and prior acceptance of established vaccines^117^^,^^118^ remain as areas for further research. The indirect benefits and long-term psychological and behavioral effects of VDMs are also noteworthy areas for further research to holistically weigh the benefits of such policies.

## Appendix Supplementary materials

Image, application 1

## Footnotes

Supplementary material associated with this article can be found in the online version at doi:10.1016/j.focus.2025.100419.
