# Supplementary material for: Impact of Vaccination-Differentiated Public Health and Social Measures on Vaccine Uptake Among the Vaccine Hesitant
Source: AJPM Focus. 2025 Aug 20;5(1):100419. doi: 10.1016/j.focus.2025.100419 (PMC12593573; doi:10.1016/j.focus.2025.100419)
Supplement: Supplementary file 1 [file mmc1.docx]

Contents

[Appendix Figure 1: Proportion of population who has received their first vaccine dose, by age group. 2](#_Toc198879543)

[Appendix Table 1: Date of vaccination rollout for each age group. 2](#_Toc198879544)

[Appendix Figure 2: Autocorrelation function plot of unadjusted baseline model. 3](#_Toc198879545)

[Appendix Figure 3: Partial autocorrelation function plot of unadjusted baseline model. 3](#_Toc198879546)

[Appendix Figure 4: Autocorrelation function plot of model adjusting for day of the week and daily numbers of COVID-19-related cases, deaths, hospitalizations, and patients in the ICU as reported by MOH. 4](#_Toc198879547)

[Appendix Figure 5: Partial autocorrelation function plot of model adjusting for day of the week and daily numbers of COVID-19-related cases, deaths, hospitalizations, and patients in the ICU as reported by MOH. 4](#_Toc198879548)

[Appendix Table 2: Announcement and implementation dates of each VDM, days between announcement and implementation, and changes in vaccination coverage 12 days before the first VDM announcement, on each VDM announcement date, and 12 days after the last VDM announcement. 5](#_Toc198879549)

[Appendix Figure 6: Number of new COVID-19 cases per day as reported by MOH. 6](#_Toc198879550)

[Appendix Figure 7: Seven-day moving average of daily COVID-19 deaths as reported by MOH. 6](#_Toc198879551)

[Appendix Figure 8: Number of cases hospitalised COVID-19 cases per day as reported by MOH. 7](#_Toc198879552)

[Appendix Figure 9: Number of COVID-19 cases in the ICU per day as reported by MOH. 7](#_Toc198879553)

[Appendix Table 3: Results of the sensitivity analyses in which distinct ITS models were fitted for each VDM announcement, describing percentage changes (95% CIs) in daily vaccine uptake rates after each VDM announcement, for the overall population. 8](#_Toc198879554)

[Appendix Table 4: Results of the sensitivity analysis using policy implementation dates instead of announcement dates as key time points for the segmented regression model, describing percentage changes (95% CIs) in daily vaccine uptake rates after each VDM announcement, for the overall population. 8](#_Toc198879555)

[Appendix Table 5: Timeline for significant policies and programs implemented in Singapore over the course of the COVID-19 pandemic. 9](#_Toc198879556)

[Appendix Table 6: Demographic makeup of the unvaccinated population 12 days before and after VDM measures were announced. 10](#_Toc198879557)


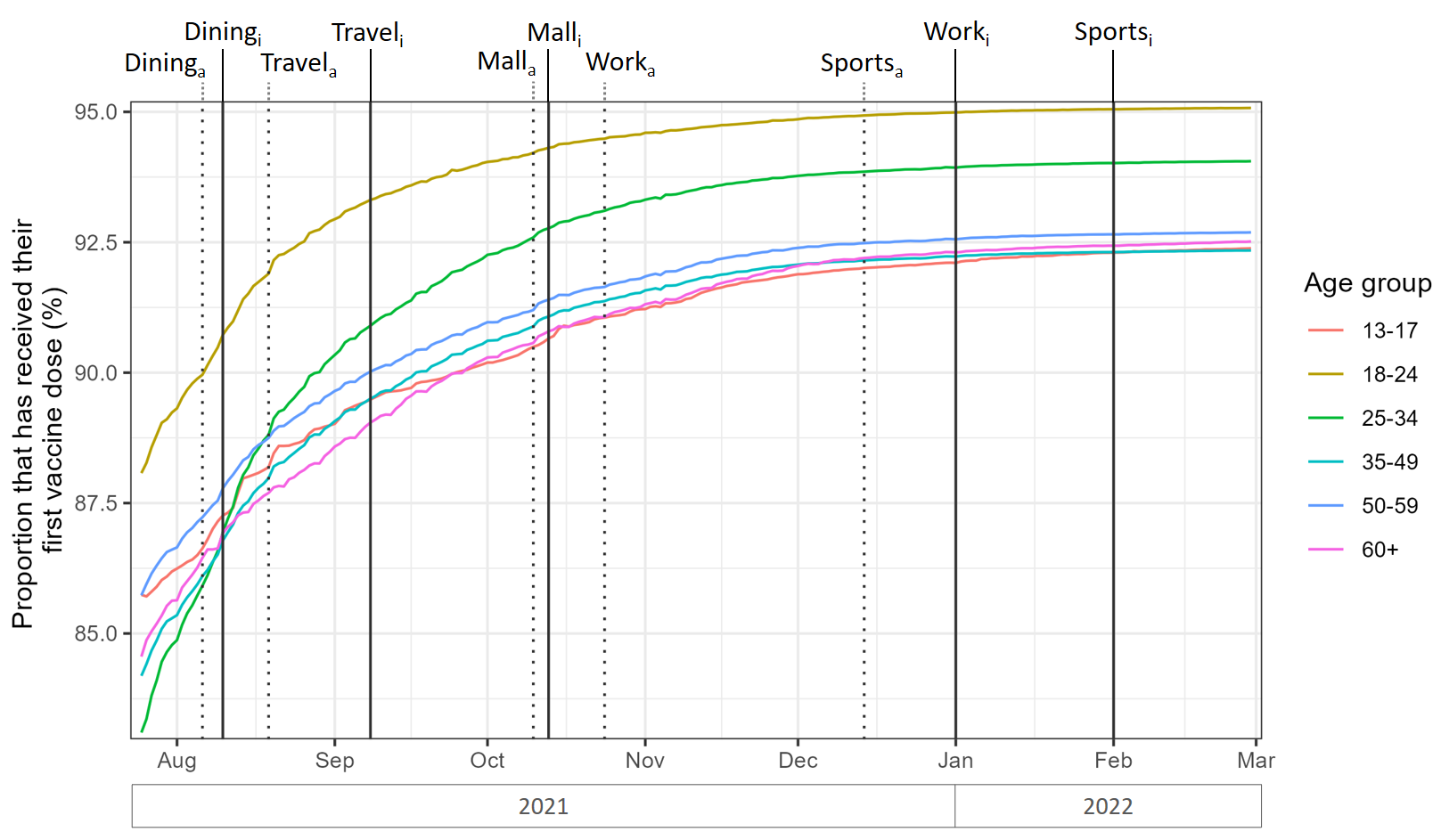


# **Appendix Figure 1:** Proportion of population who has received their first vaccine dose, by age group.

*Note.* For each VDM, announcement dates were indicated by subscript “a” and dotted vertical lines, while implementation dates were indicated by subscript “i” and solid vertical lines.

| Date | Age group |
| --- | --- |
| 22 Feb 2021 | > 70 years old |
| 8 Mar 2021 | 60 - 69 years old |
| 24 Mar 2021 | 45 - 59 years old |
| 19 May 2021 | 40 - 44 years old |
| 1 Jun 2021 | > 12 years old (Priority given to those taking national exams) |

# **Appendix Table 1:** Date of vaccination rollout for each age group.


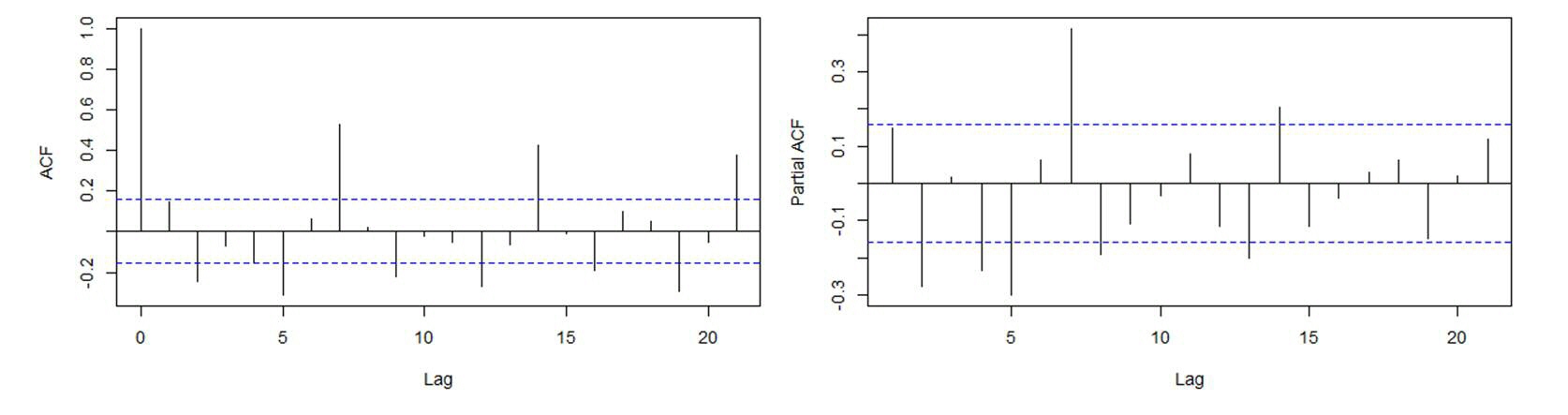


# Appendix Figure 2: Autocorrelation function plot of unadjusted baseline model.


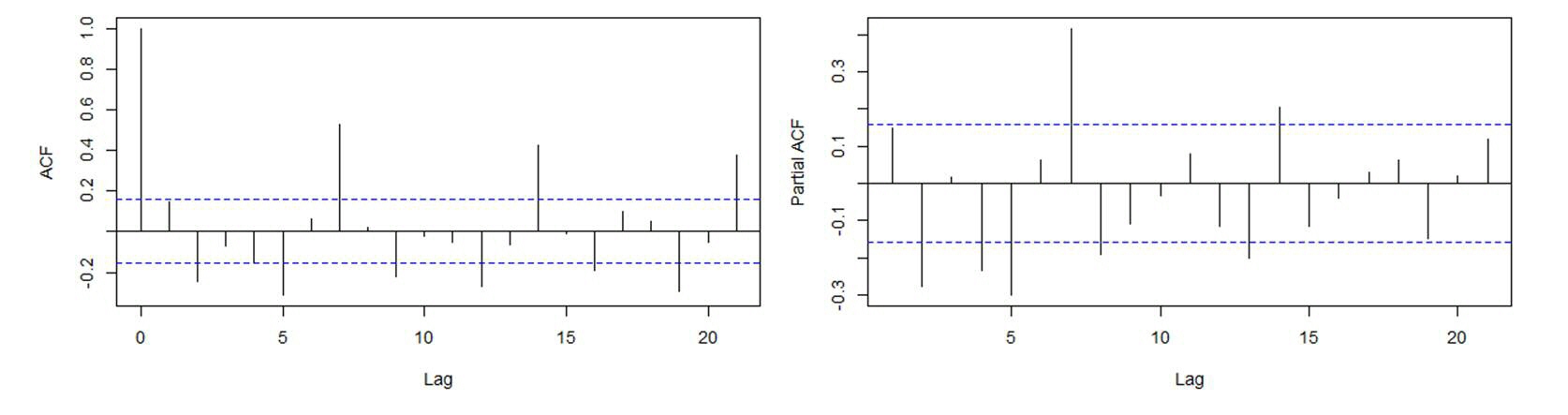


# Appendix Figure 3: Partial autocorrelation function plot of unadjusted baseline model.


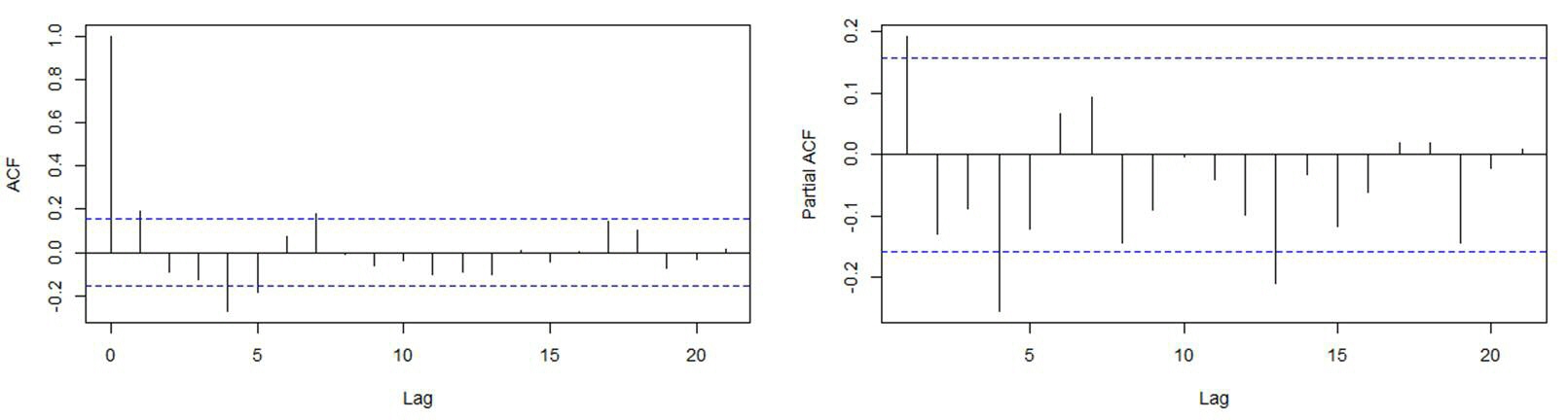


# Appendix Figure 4: Autocorrelation function plot of model adjusting for day of the week and daily numbers of COVID-19-related cases, deaths, hospitalizations, and patients in the ICU as reported by MOH.


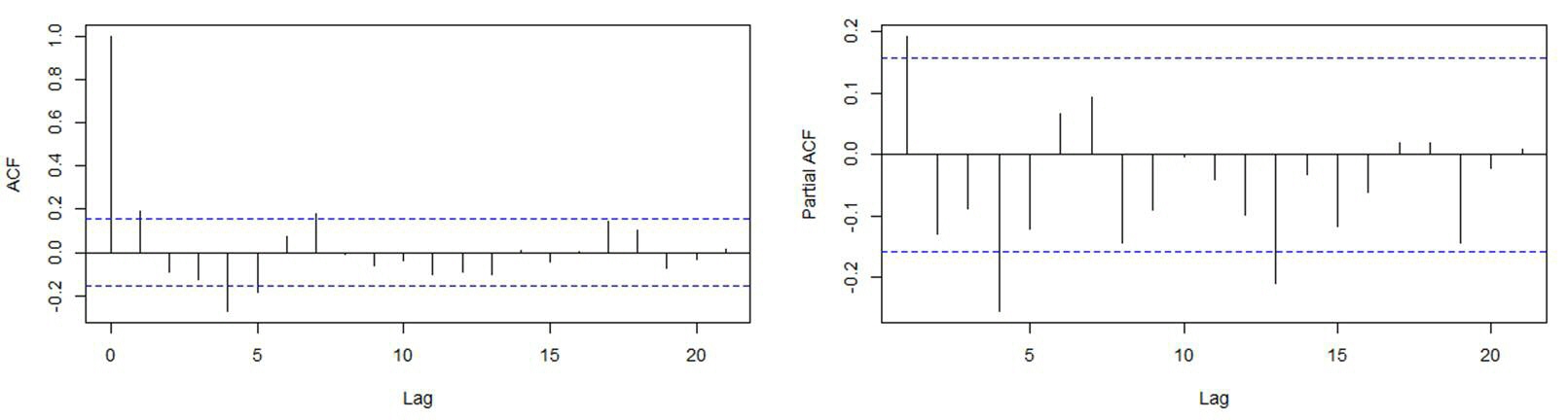


# Appendix Figure 5: Partial autocorrelation function plot of model adjusting for day of the week and daily numbers of COVID-19-related cases, deaths, hospitalizations, and patients in the ICU as reported by MOH.

| Vaccine-differentiated measure (VDM) | | Date | Days between | Percentage of population that was vaccine-naive on given date | Percentage change in percentage of vaccine-naive individuals since previous date | Number of individuals who received their dose since previous date | Percentage of population that has received first dose on given date |
| --- | --- | --- | --- | --- | --- | --- | --- |
| 12 days before first VDM announcement | | 25 Jul 2021 |  | 15.2 |  |  | 84.8 |
| Dining | Announcement | 6 Aug 2021 | 4 | 13.3 | 1.90 | 78,074 | 86.7 |
|  | Implementation | 10 Aug 2021 |  |  |  |  |  |
| Travel | Announcement | 19 Aug 2021 | 20 | 9.4 | 3.90 | 156,990 | 90.6 |
|  | Implementation | 8 Sep 2021 |  |  |  |  |  |
| Shopping malls | Announcement | 10 Oct 2021 | 3 | 8.6 | 0.80 | 35,211 | 91.4 |
|  | Implementation | 13 Oct 2021 |  |  |  |  |  |
| Work | Announcement | 23 Oct 2021 | 70 | 8.1 | 0.50 | 18,961 | 91.9 |
|  | Implementation | 1 Jan 2022 |  |  |  |  |  |
| Sports/IHLs/Hotels | Announcement | 14 Dec 2021 | 49 | 7.3 | 0.80 | 35,911 | 92.7 |
|  | Implementation | 1 Feb 2022 |  |  |  |  |  |
| 12 days after last VDM announcement | | 26 Dec 2021 |  | 7.2 | 0.10 | 2,196 | 92.8 |

# Appendix Table 2: Announcement and implementation dates of each VDM, days between announcement and implementation, and changes in vaccination coverage 12 days before the first VDM announcement, on each VDM announcement date, and 12 days after the last VDM announcement.


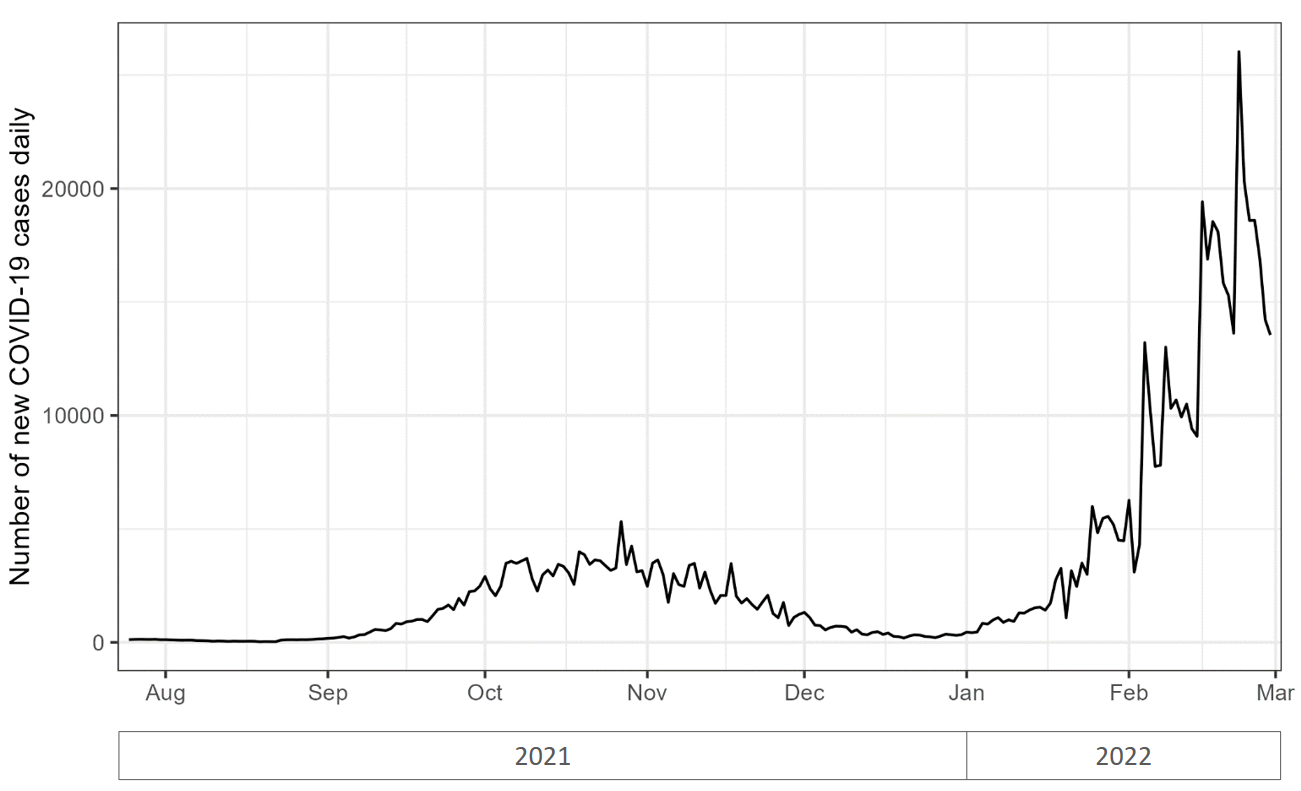


# Appendix Figure 6: Number of new COVID-19 cases per day as reported by MOH.


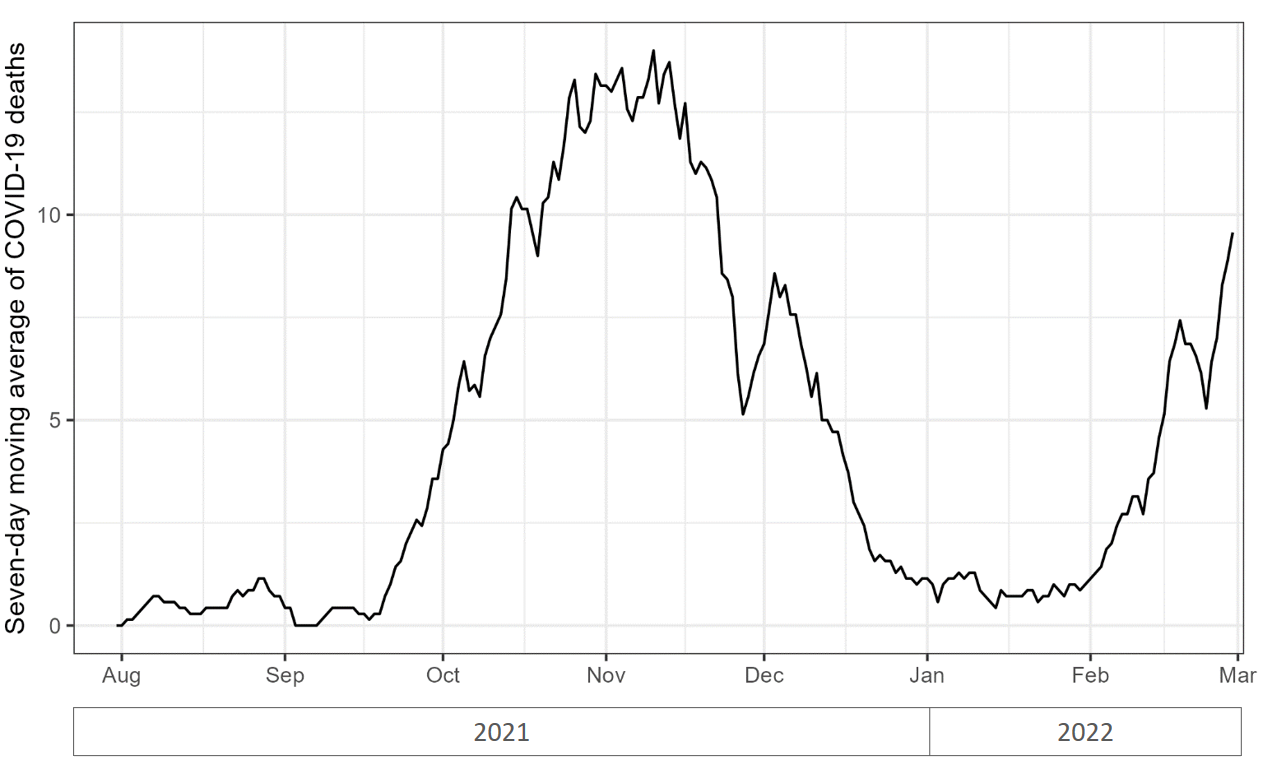


# Appendix Figure 7: Seven-day moving average of daily COVID-19 deaths as reported by MOH.


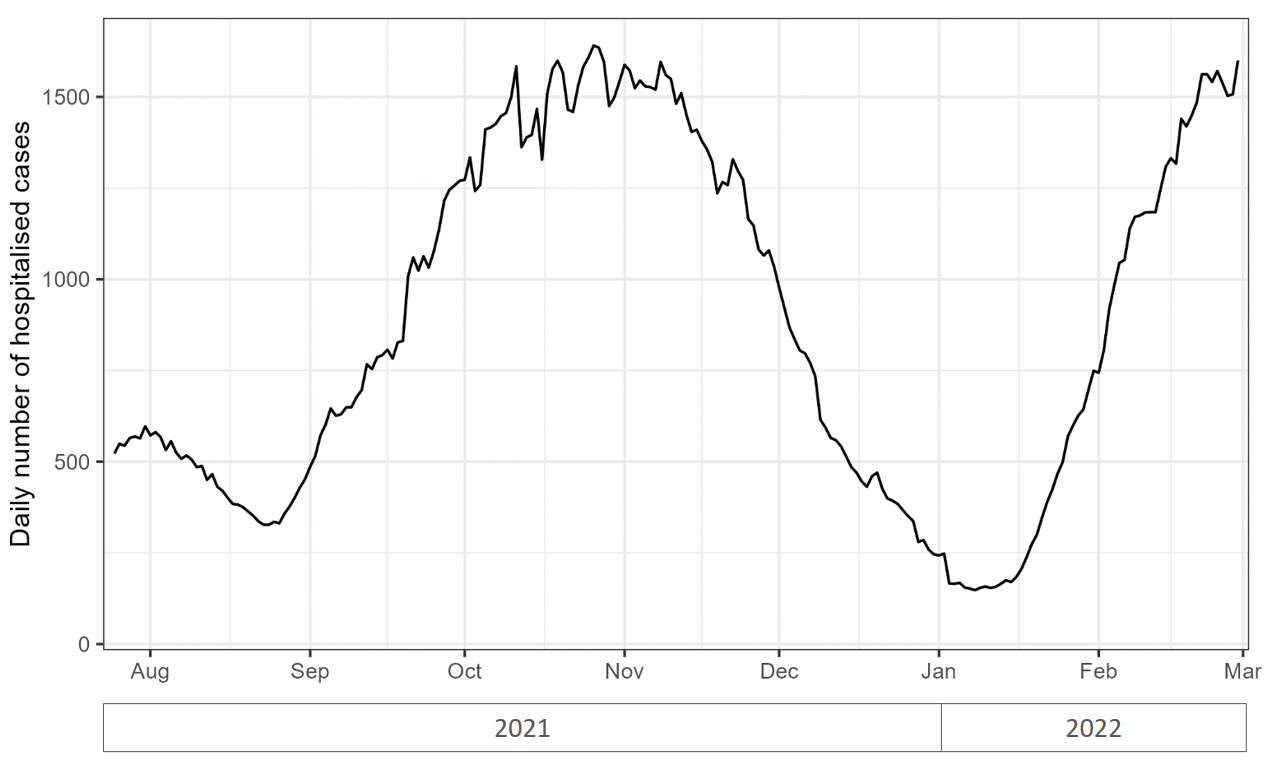


# Appendix Figure 8: Number of cases hospitalised COVID-19 cases per day as reported by MOH.


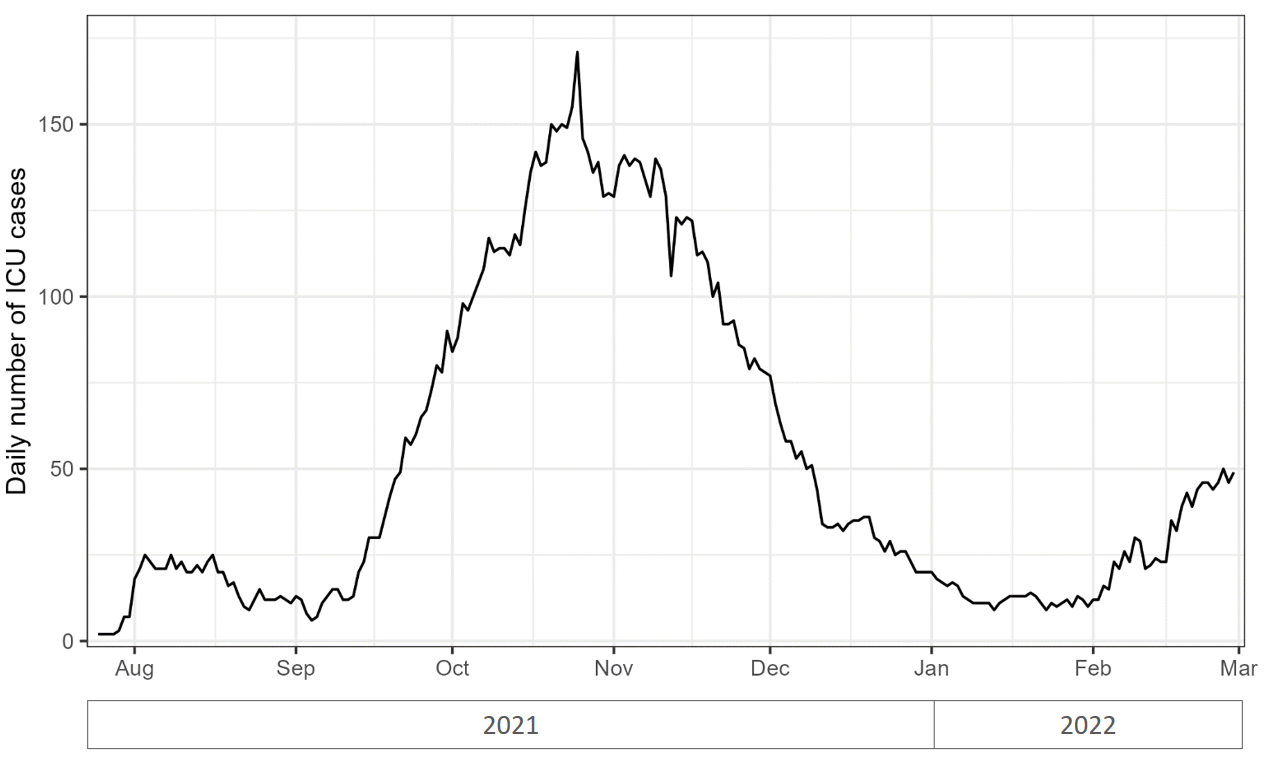


# Appendix Figure 9: Number of COVID-19 cases in the ICU per day as reported by MOH.

*Note.* We observe an increase in hospitalised and ICU cases in September 2021, as well as an increase in new cases and deaths in mid-September 2021, with numbers peaking in October-November 2021. These trends could have partially contributed to the uptick in vaccination observed in mid-October 2021 as seen in Figure 1. Nonetheless, sensitivity analyses accounting for trends in Covid-19 cases, deaths, hospitalisations, and ICU cases (Table 2) led to similar findings as the main model.

| Model | Dining | Travel | Malls | Work | Sports/IHLs/Hotels |
| --- | --- | --- | --- | --- | --- |
| Single model per event | 0.41 (-0.06, 0.87) | -0.18 (-0.49, 0.13) | 0.33 (0.21, 0.45)*** | 0.11 (-0.06, 0.27) | 0.04 (0.01, 0.07)** |
| Single model per event, adjusted† | -0.02 (-0.23, 0.18) | -0.15 (-0.29, -0.00)* | 0.38 (0.25, 0.51)*** | -0.04 (-0.09, 0.01) | -0.02 (-0.07, 0.03) |

# Appendix Table 3: Results of the sensitivity analyses in which distinct ITS models were fitted for each VDM announcement, describing percentage changes (95% CIs) in daily vaccine uptake rates after each VDM announcement, for the overall population.

**p* < .05, ***p* < .01, ****p* < .001

† Adjusted for day of the week, daily numbers of COVID-19-related cases, deaths, hospitalizations, and patients in the ICU as reported by MOH

*Note.* These sensitivity analyses were conducted to independently assess the effect of each VDM announcement. The data points for the regression analysis included observations from 12 days before and 12 days after the announcement. As vaccines were highly accessible and administered on a walk-in basis during the time of VDM announcements, the 12-day time period was deemed a sufficient window for interested persons to obtain vaccination. This also provided sufficient time points to monitor vaccination rates before, during, and after the VDM announcements. Results were consistent with findings from the main model.

| Model | Dining | Travel | Malls | Work | Sports/IHLs/Hotels |
| --- | --- | --- | --- | --- | --- |
| Segmented regression  (main model) | 0.36 (0.17, 0.55)*** | 0.13 (-0.05, 0.30) | -0.04 (-0.18, 0.10) | 0.05 (-0.00, 0.10) | -0.01 (-0.05, 0.03) |

# Appendix Table 4: Results of the sensitivity analysis using policy implementation dates instead of announcement dates as key time points for the segmented regression model, describing percentage changes (95% CIs) in daily vaccine uptake rates after each VDM announcement, for the overall population.

**p* < .05, ***p* < .01, ****p* < .001

*Note.* Changes in vaccine uptake rate were largely driven by policy announcements rather than policy implementation.

| Date | Policy/Program |
| --- | --- |
| 30/12/2020 | Vaccination of healthcare workers and COVID-19 frontline workers |
| 8/1/2021 | Prime Minister and Director of Medical Services receive first dose of Pfizer-BioNTech COVID-19 vaccine |
| 13/2/2021 | COVID-19 vaccination available at all public polyclinics |
| 18/2/2021 | Vaccination of persons ages 70 years and above |
| 8/3/2021 | Progressive lowering of eligible age groups for vaccination |
| 17/3/2021 | Launch of Vaccine Injury Financial Assistance Programme for COVID-19 Vaccination (VIFAP) |
| 18/5/2021 | Addition of 40 vaccination centers and 60 Public Health Preparedness Clinics(PHPCs). |
| 31/5/2021 | Vaccination for paediatrics ages 12 to 18 years |
| 1/6/2021 | Walk-in vaccination for persons ages 60 years and above |
| 29/6/2021 | Daily reporting on COVID-19 case and death counts |
| 15/7/2021 | Launch of home vaccination programme |
| 6/8/2021 | Announcement of VDM for dining |
| 10/8/2021 | Implementation of VDM for dining |
| 13/8/2021 | "Let’s Get Our Seniors Vaccinated" programme, $30 voucher per senior referred |
| 19/8/2021 | Announcement of VDM for travel |
| 8/9/2021 | Implementation of VDM for travel |
| 10/12/2021 | Announcement of VDM for shopping malls |
| 13/12/2021 | Implementation of VDM for shopping malls |
| 23/10/2021 | Announcement of VDM for work |
| 14/12/2021 | Announcement of VDM for Sports/IHLs/Hotels |
| 27/12/2021 | Introduction of vaccination for children ages 5 to 11 years |
| 1/1/2022 | Implementation of VDM for work |
| 1/2/2022 | Implementation of VDM for Sports/IHLs/Hotels |
| 10/10/2022 | All vaccination-differentiated measures are lifted |

# Appendix Table 5: Timeline for significant policies and programs implemented in Singapore over the course of the COVID-19 pandemic.

| Demographic | Characteristics | 12 days before first VDM (%) | 12 days after last VDM (%) | h* |
| --- | --- | --- | --- | --- |
| Sex | Female | 55.97 | 56.90 | -0.019 |
|  | Male | 44.03 | 43.10 | 0.019 |
| Ethnicity | Chinese | 70.18 | 67.93 | 0.049 |
|  | Indian | 12.50 | 15.60 | -0.089 |
|  | Malay | 12.37 | 8.92 | 0.112 |
|  | Others | 4.95 | 7.54 | -0.108 |
| Housing type | 1-2 Room | 6.24 | 4.05 | 0.100 |
|  | 3-Room | 15.90 | 14.17 | 0.048 |
|  | 4-Room | 27.81 | 21.59 | 0.144 |
|  | 5-Room/Executive | 31.06 | 26.25 | 0.106 |
|  | Private | 9.59 | 14.04 | -0.302 |
|  | Others | 9.40 | 19.90 | -0.138 |
| Age group | 13-17 | 5.47 | 6.76 | -0.054 |
|  | 18-24 | 7.13 | 6.12 | 0.041 |
|  | 25-34 | 19.14 | 13.89 | 0.142 |
|  | 35-49 | 25.73 | 26.57 | -0.019 |
|  | 50-59 | 15.17 | 17.06 | -0.051 |
|  | 60+ | 27.37 | 29.60 | -0.049 |
| Total |  | 603,717 | 265,739 |  |

# Appendix Table 6: Demographic makeup of the unvaccinated population 12 days before and after VDM measures were announced.

*Note.* *Cohen’s test h was performed, a test for proportions using effect size. (h = 0.2 is indicative of a small effect, h = 0.5 of medium effect and h = 0.8 of a large effect)
